# Advantages of InGaN–GaN–InGaN Delta Barriers for InGaN-Based Laser Diodes

**DOI:** 10.3390/nano11082070

**Published:** 2021-08-15

**Authors:** Liwen Cheng, Zhenwei Li, Jiayi Zhang, Xingyu Lin, Da Yang, Haitao Chen, Shudong Wu, Shun Yao

**Affiliations:** 1 College of Physical Science and Technology, Yangzhou University, Yangzhou 225002, China; zhenweiliyzu@gmail.com (Z.L.); zhangjiayi936@gmail.com (J.Z.); yzujslxy@gmail.com (X.L.); yangdayzu@gmail.com (D.Y.); htchen@yzu.edu.cn (H.C.); sdwu@yzu.edu.cn (S.W.); 2 Sino-Semiconductors Technologies Co., Ltd., Taizhou 225300, China; yaoshun@sinosemic.com

**Keywords:** InGaN–GaN–InGaN delta barriers, InGaN, laser diodes, electron leakage, hole injection

## Abstract

An InGaN laser diode with InGaN–GaN–InGaN delta barriers was designed and investigated numerically. The laser power–current–voltage performance curves, carrier concentrations, current distributions, energy band structures, and non-radiative and stimulated recombination rates in the quantum wells were characterized. The simulations indicate that an InGaN laser diode with InGaN–GaN–InGaN delta barriers has a lower turn-on current, a higher laser power, and a higher slope efficiency than those with InGaN or conventional GaN barriers. These improvements originate from modified energy bands of the laser diodes with InGaN–GaN–InGaN delta barriers, which can suppress electron leakage out of, and enhance hole injection into, the active region.

## 1. Introduction

GaN-based laser diodes (LDs) have widespread applications in data storage, laser-based TVs, and mobile laser projectors [1,2,3]. High-performance GaN LDs are promising new laser sources for medical imaging, high-density optical storage, solid-state lighting, and full-color displays [4,5,6].

Many efforts have been made to improve the performance of GaN LDs, such as increased output power, decreased threshold current, and enhanced slope efficiency. Improved designs include various electron blocking layers (EBLs) for better suppression of electron leakage [7,8,9] and optimization of various waveguide layers (WLs) and cladding layers (CLs) to decrease internal optical absorption losses [10,11,12]. There has been less research on the multiple quantum well (MQW) active region of GaN LDs relative to that on GaN-based light-emitting diodes (LEDs) because the LD structure is much more complex. Recently, Yang et al. reported that using InGaN barriers to replace the conventional GaN barriers can reduce optical field leakage because InGaN has a higher refractive index than does GaN [13]. In addition, Kuo et al. reported that InGaN barriers in GaN-based LEDs can reduce the polarization effect in the MQWs [14]. Additionally, the improved LED performance can be obtained because of the smaller lattice mismatch between InGaN barriers and InGaN QWs than that between conventional GaN barriers and InGaN QWs [14]. However, because the InGaN energy bandgap is smaller than that of GaN, InGaN barriers will reduce carrier confinement in the MQWs.

Here, an LD with InGaN–GaN–InGaN delta (IGID) barriers is designed to decrease the polarization effect without reducing carrier confinement in the MQWs. The optoelectronic properties and device performance of these LDs are numerically compared with those with GaN and InGaN barriers. The simulations are performed with LASTIP software [15], which is frequently used to predict the characteristics of GaN-based LDs [16,17,18].

## 2. Structures and Parameters

**Figure 1:**
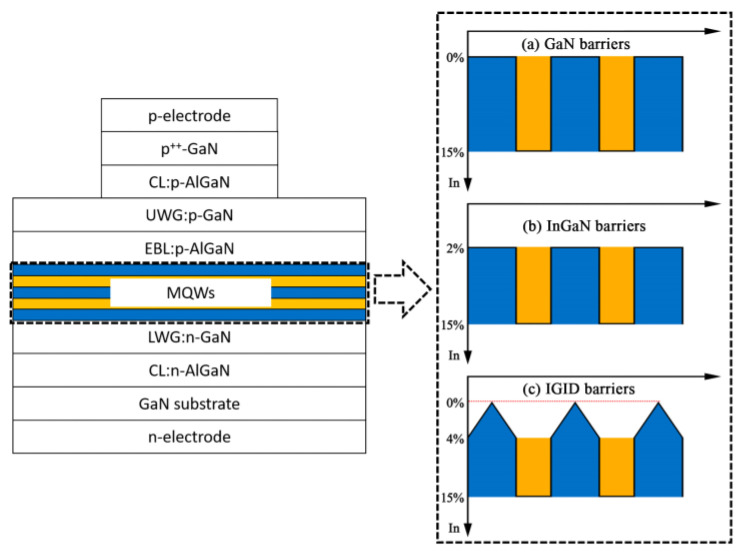
Schematics of LD structures with (**a**) GaN, (**b**) InGaN, and (**c**) IGID barriers.

In the simulations, InGaN LDs with GaN barriers were based on Ref [16], and consist of a 1 μm Si-doped (3 × 10^18^ cm^−3^) c-plane GaN substrate, a 1 μm Si-doped (3 × 10^18^ cm^−3^) Al_0.08_Ga_0.92_N CL, and a 0.12 μm Si-doped (3 × 10^18^ cm^−3^) GaN lower WL. The MQWs consisted of two 2.5 nm In_0.15_Ga_0.85_N QWs and three 14 nm GaN barriers. On top of the MQWs was a 20 nm Mg-doped (5 × 10^19^ cm^−3^) Al_0.15_Ga_0.85_N EBL, a 0.1 μm Mg-doped (2 × 10^19^ cm^−3^) GaN upper WL, a 0.6 μm Mg-doped (2 × 10^19^ cm^−3^) Al_0.06_Ga_0.94_N CL, and a 40 nm Mg-doped (1 × 10^20^ cm^−3^) GaN ohmic contact layer. The LDs with InGaN and IGID barriers had identical structures as described above, except that the GaN barriers were replaced with In_0.02_Ga_0.98_N and IGID barriers, respectively. For the IGID barriers, the indium content of the InGaN barriers was gradually decreased from 0.04 to 0 and then increased to 0.04 along the growth direction. The three types of LD barriers are shown schematically in Figure 1, where the IGID barriers formed delta shapes. The overall indium content in the InGaN and IGID barriers was the same. The ridge widths and cavity lengths of the three LDs were 3 µm and 600 µm, respectively.

In the simulations, the intrinsic interface charge from piezoelectric and spontaneous polarizations was calculated via approaches reported by Fiorentini et al. [19,20], and the screen factor was set to 0.25. The Auger recombination coefficient and the Shockley–Read–Hall (SRH) carrier lifetime were assumed to be 2.0 × 10^−30^ cm^6^ s^−1^ and 100 ns, respectively. The absorption coefficient of each layer in the LDs was set to be proportional to the doping concentration, as given by:

(1) $$\mathrm{absorption}\;\mathrm{coefficient}=\frac{\mathrm{doping}\;\mathrm{concentration}\;({\mathrm{cm}}^{-3})}{{\mathrm{10}}^{19}\;({\mathrm{cm}}^{-3})}\times 50\;({\mathrm{cm}}^{-1})$$

The refractive indices of AlGaN and InGaN were estimated according to Chen et al. [21]. Other material parameters used in the simulations were based on those reported in Ref [22].

## 3. Results

**Figure 2:**
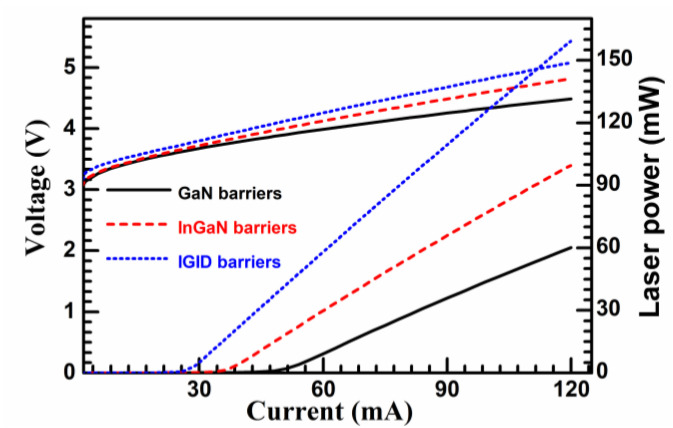
The laser power vs. current–voltage (L–I–V) characteristic curves for the three LD structures.

Figure 2 plots the laser power vs. current–voltage (L–I–V) characteristic curves for the three LD structures. In the I–V curves, the LDs have very similar same turn-on voltages, but the resistance of the LD with IGID barriers was higher than that of the LD with InGaN barriers, which, in turn, was higher than that of the LD with GaN barriers. For the L–I curves, the threshold currents were 49.7 mA, 36.2 mA, and 25.7 mA, and the laser powers were 59.8 mW, 100.0 mW, and 159.8 mW at 120 mA, for the LDs with GaN, InGaN, and IGID barriers, respectively. In addition, the LD with IGID barriers exhibited the highest slope efficiency at 1.69 W/A. The slope efficiency of the LD with GaN barriers was 0.76 W/A, and that for the LD with InGaN barriers was 1.14 W/A. Thus, the LD with IGID barriers had the lowest threshold current, the highest slope efficiency, and the highest laser power, which indicate that it had the best performance.

**Figure 3:**
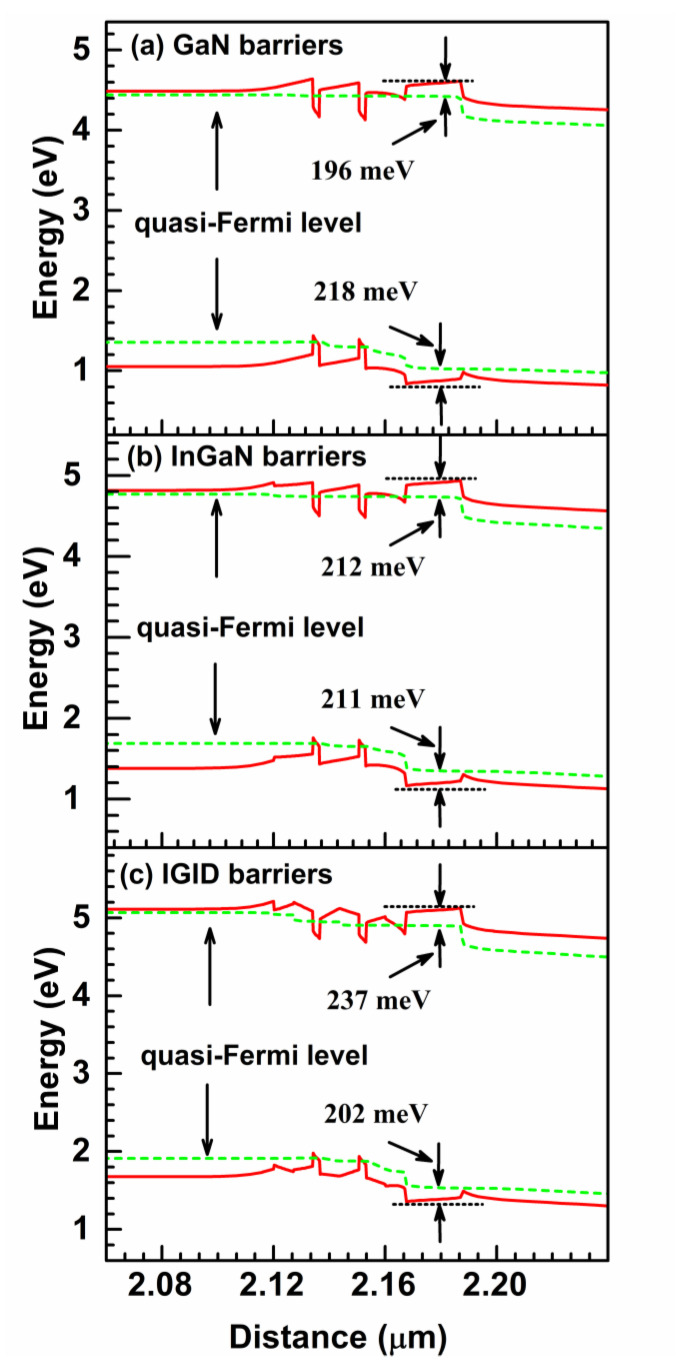
Energy band diagrams for the LDs with the (**a**) GaN, (**b**) InGaN, and (**c**) IGID barriers at 120 mA.

Figure 3 shows energy band diagrams for the LDs with the GaN, InGaN, and IGID barriers at 120 mA. In Figure 3a, the energy band for the LD with GaN barriers exhibits significant tilting because of the strong polarization effect. A potential conduction barrier for electrons was formed at the interface between the last barrier (LB) near the *p*-side and the EBL. This was because of the difference in the energy gap between the GaN LB and the AlGaN EBL, which can block electron leakage. A potential barrier for holes was also formed in the valence band, which can impede hole injection from the *p*-side into the MQW region. In addition, because of the polarization effect, the LB tilts downward while the EBL tilts upward. Therefore, a triangular potential well was formed in the conduction band at this interface, which tends to confine electrons. Due to the lower conduction band energy of the InGaN LB relative to that of the GaN LB, as well as the larger polarization field at the LB/EBL interface, the effective barrier height for electrons increased from 196 meV to 212 meV when the InGaN barriers were substituted for the GaN barriers (see Figure 3b). Additionally, the depth of the triangular potential well at the LB/EBL interface increased. Most of the electrons are confined there, which suppresses electron leakage into the p-region. Moreover, the effective barrier height for holes was reduced from 218 meV to 211 meV because of the increased valence band energy of the LB. Hence, the efficiency of hole injection should be enhanced.

As illustrated in Figure 3c, the energy band diagram of the LD with IGID barriers exhibited the best performance. The effective barrier height of the EBL for electrons was increased to 237 meV and that for holes was reduced to 202 meV. The triangular potential well at the LB/EBL interface had the maximum depth among the three LDs, and another potential well was formed in the conduction band at the n-GaN WL/first barrier interface near the n-side. Thus, most electrons could be confined. Meanwhile, the valence band edge of the LB near the LB/EBL interface was even lower than the quasi-Fermi level of the p-GaN waveguide layer. Hence, a potential well for holes in the LB was formed. This indicates that more holes could tunnel into the MQW region from *p*-side through this well.

**Figure 4:**
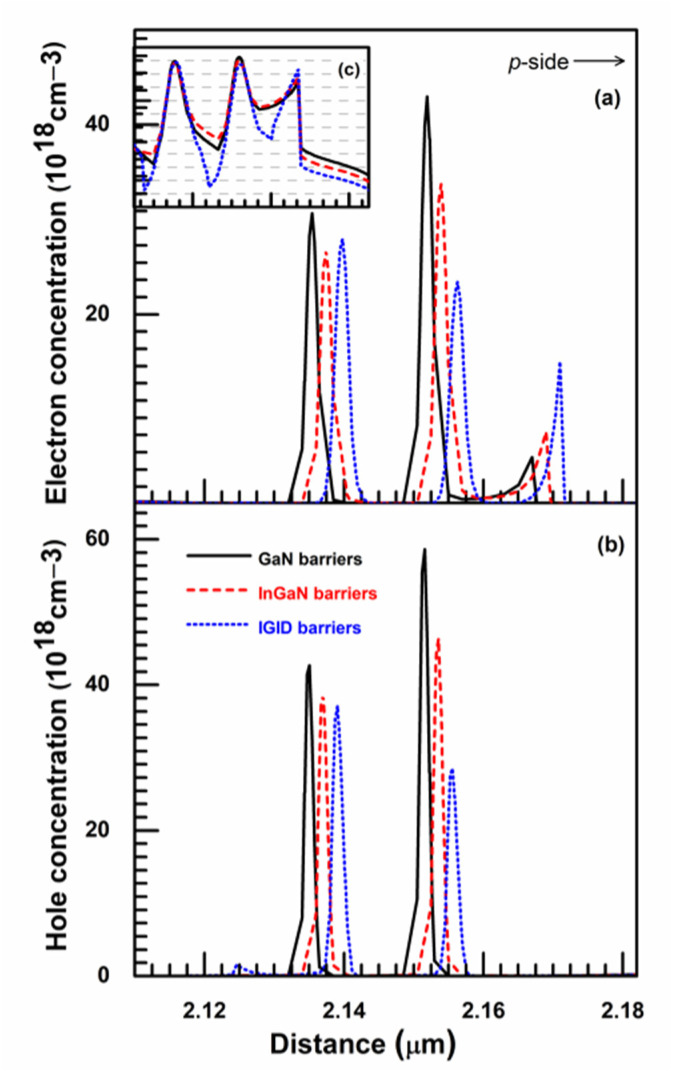
The electron and hole concentration distributions for the (**a**) GaN, (**b**) InGaN, and (**c**) IGID LDs at 120 mA.

The electron and hole concentration distributions for the three LDs at 120 mA are shown in Figure 4. For convenient visual analysis, the horizontal coordinate of the LDs with InGaN and IGID barriers were slightly moved in the diagrams. The electron and hole concentrations in the MQWs of the LDs with GaN barriers were the highest, whereas those in the MQWs of the LDs with IGID barriers were the lowest. In contrast to the LEDs, the carrier concentrations in the LD MQWs were almost saturated and remained constant when the current was injected over the threshold current. These are “transparency” carrier concentrations. With lower transparency carrier concentrations and a lower threshold current, a higher laser power is possible because of increased injected carriers that participate in stimulated recombinations. This is consistent with the analysis of the L–I curves above. In addition, the number of electrons confined at the LB/EBL interface increased in the LD with IGID barriers, indicating decreased electron leakage into the *p*-side. This was verified in the inset of Figure 4a, which plots the electron distributions on a logarithmic scale. Electron leakage to the *p*-side decreased substantially in the LD with IGID barriers relative to that in the other LDs. Moreover, from the inset of Figure 4a, it can be clearly observed that the electron concentrations in the barriers of the LD with IGID barriers were much smaller than those in the LDs with GaN and InGaN barriers, which indicated that it had the best electron confinement in the QWs.

**Figure 5:**
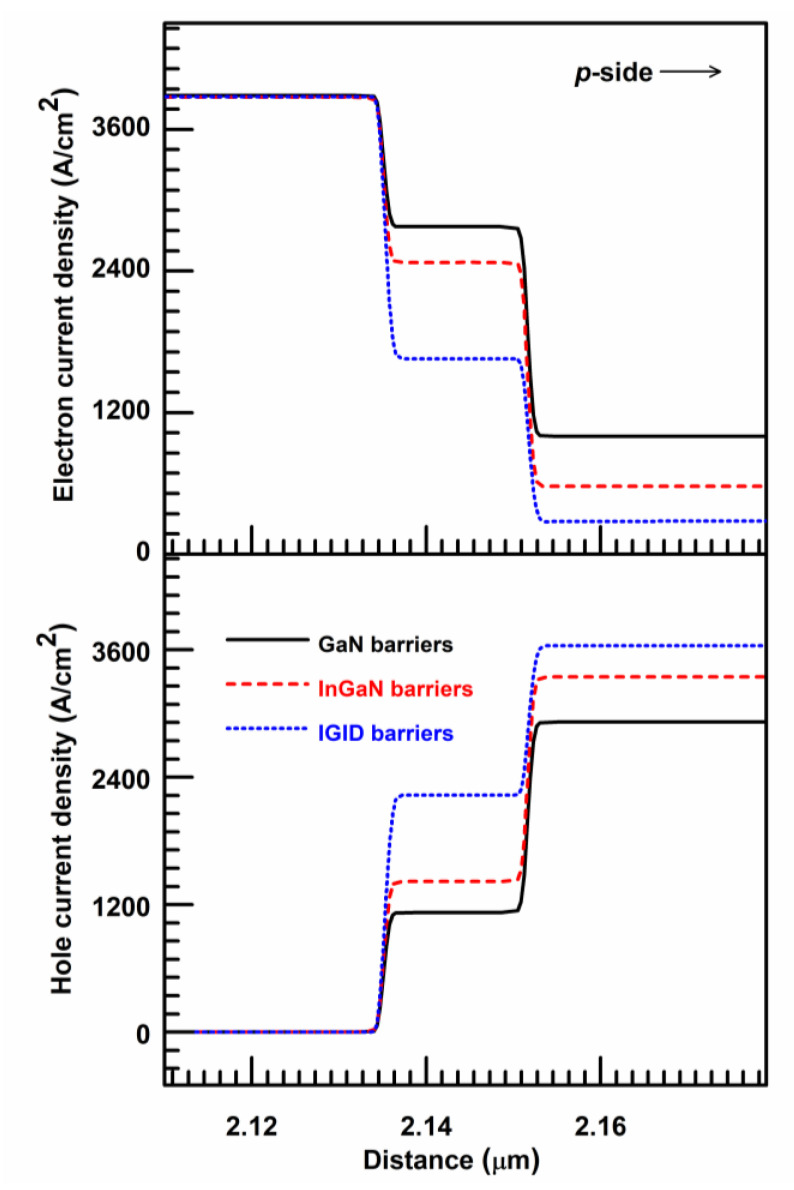
The electron and hole current density distributions in the LDs with (a) GaN, (b) InGaN, and (c) IGID barriers at 120 mA.

Figure 5 displays the electron and hole current density distributions in the LDs with GaN, InGaN, and IGID barriers at 120 mA. The electrons and holes, respectively, were injected into the MQW region from the n-side and the *p*-side and were then recombined in the QWs. Thus, both the electron current density from the n-side to the *p*-side and the hole current density from the *p*-side to n-side along the QWs decreased. The electrons and holes that, respectively, leak into the *p*-side and n-side from the MQW region will cause electron and hole leakage currents, and these leakage carriers do not participate in stimulated recombinations. The LD with GaN barriers had a large electron leakage current of 1322.0 A/cm^2^. For the LDs with InGaN and IGID barriers, the electron leakage currents were well suppressed, with values of 594.5 A/cm^2^ and 280.5 A/cm^2^, respectively. There was very little hole leakage current at nearly 0 A/cm^2^. However, the injected hole current densities were 2613.8 A/cm^2^, 3337.2 A/cm^2^, and 3649.6 A/cm^2^ for the LDs with GaN, InGaN, and IGID barriers, respectively. Hence, the hole injection efficiency can be significantly enhanced in the LD with IGID barriers.

**Figure 6:**
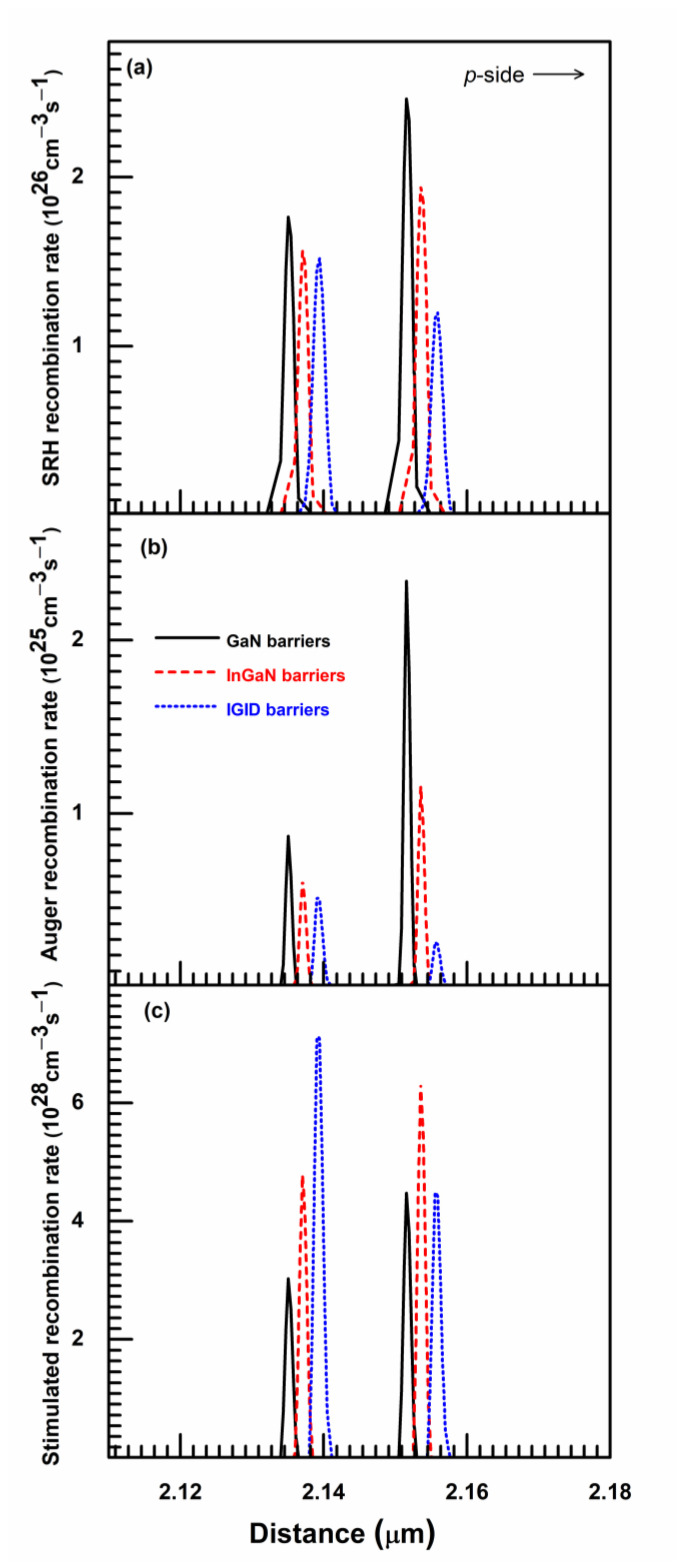
The SRH, Auger, and stimulated recombination rates for the (**a**) GaN, (**b**) InGaN, and (**c**) IGID LDs.

The SRH, Auger, and stimulated recombination rates for the three LDs are depicted in Figure 6. The SRH and Auger recombinations are mainly non-radiative processes in the MQWs, where the rates are proportional to the first and third powers of carrier concentrations, respectively. As the MQWs for the LD with IGID barriers had the lowest transparency carrier concentrations, the SRH and Auger recombination rates in the active region decreased (Figure 6a). In addition, those LDs had the best electron confinement in the QWs and the highest hole injection efficiency, as discussed above. Thus, many MQW carriers participate in stimulated recombinations, increasing the overall recombination rates in the LDs with IGID barriers by 62.6% and 5.2%, relative to those LDs with GaN and InGaN barriers, respectively. Hence, the optical performance of the LD with IGID barriers improved markedly, and the stimulated recombination rate in the QW near the n-side was larger than that in the QW near the *p*-side. The latter was mainly because the modified valence band configuration of the LD with IGID barriers, as shown in Figure 3, is favorable to the hole injection. It allows most holes to inject into the QW near the n-side and to participate in stimulated recombinations.

## 4. Conclusions

In conclusion, the optical and electronic characteristics of GaN-based LDs with GaN, InGaN, and IGID barriers were studied systematically via simulations. The results suggested that the LD with IGID barriers exhibited the best performance with a relatively low turn-on current, a higher laser power, and a higher slope efficiency. The major physical origin of these improvements was the properly adjusted energy bands that reduce the leakage of electrons and improve hole injections into the active region.
